# Cystinosis: Diagnostic Role of Bone Marrow Examination

**DOI:** 10.4274/Tjh.012.0194

**Published:** 2014-03-05

**Authors:** Shahla Ansari, Ghasem Miri Aliabad, Yousefian Saeed

**Affiliations:** 1 Tehran University of Medical Sciences, Department of Pediatric Hematology-Oncology, Tehran, Iran

**Keywords:** Cystinosis, Bone marrow, Examination

The patient was a 1.5-year-old boy who presented with growth failure, inability to walk, polyuria, and polydipsia. On physical examination, he had fair skin and blond hair. He did not have organomegaly. Ophthalmologic examination by an ophthalmologist was normal. Hypophosphatemia, hypovitaminosis D, mild acidosis, glucosuria, proteinuria, and generalized aminoaciduria were found upon laboratory studies. He underwent bone marrow aspiration that revealed increased numbers of macrophages containing polygonal crystals. 

Severe and widespread deposition of crystals in bone marrow particles (Figure 1) was observed, which was pathognomonic for cystinosis. Cystinosis is a rare autosomal recessive disorder caused by a defect in cystine transport outside the lysosomes, leading to accumulation of cystine crystals in various organs including the kidneys, liver, eyes, and brain [1]. It has 3 forms and infantile nephrophatic cystinosis is the most common and most severe type. Clinical manifestations of this variant include polyuria, polydipsia, dehydration, acidosis, rickets, and failure to thrive due to tubular dysfunction and Fanconi’s syndrome [1,2]. The diagnosis is made by observing corneal cystine crystals and/or measuring the cystine content of leukocytes [1]; however, typical crystals in the bone marrow are diagnostic. Early diagnosis and treatment of these patients can prevent kidney function impairment and other complications secondary to deposition of cystine crystals in various tissues. Informed consent was obtained.

## CONFLICT OF INTEREST STATEMENT

The authors of this paper have no conflicts of interest, including specific financial interests, relationships, and/ or affiliations relevant to the subject matter or materials included.

## Figures and Tables

**Figure 1:**
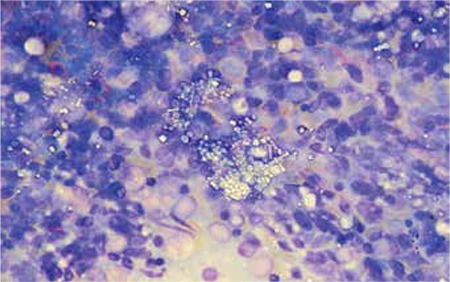
Widespread deposition of crystals in bone marrow fragment.
